# Increased water temperature and turbidity act independently to alter social behavior in guppies (*Poecilia reticulata*)

**DOI:** 10.1002/ece3.9958

**Published:** 2023-03-28

**Authors:** Imranah Allibhai, Costanza Zanghi, Martin J. How, Christos C. Ioannou

**Affiliations:** ^1^ School of Biological Sciences University of Bristol Bristol UK

**Keywords:** aggregation, collective behavior, environmental change, multiple stressors, refuge use, shoaling

## Abstract

Changes in environmental conditions can shift the costs and benefits of aggregation or interfere with the sensory perception of near neighbors. This affects group cohesion with potential impacts on the benefits of collective behavior such as reduced predation risk. Organisms are rarely exposed to one stressor in isolation, yet there are only a few studies exploring the interactions between multiple stressors and their effects on social behavior. Here, we tested the effects of increased water temperature and turbidity on refuge use and three measures of aggregation in guppies (*Poecilia reticulata*), increasing temperature and turbidity in isolation or in combination. When stressors were elevated in isolation, the distribution of fish within the arena as measured by the index of dispersion became more aggregated at higher temperatures but less aggregated when turbidity was increased. Another measure of cohesion at the global scale, the mean inter‐individual distance, also indicated that fish were less aggregated in turbid water. This is likely due to turbidity acting as a visual constraint, as there was no evidence of a change in risk perception as refuge use was not affected by turbidity. Fish decreased refuge use and were closer to their nearest neighbor at higher temperatures. However, the nearest neighbor distance was not affected by turbidity, suggesting that local‐scale interactions can be robust to the moderate increase in turbidity used here (5 NTU) compared with other studies that show a decline in shoal cohesion at higher turbidity (>100 NTU). We did not observe any significant interaction terms between the two stressors, indicating no synergistic or antagonistic effects. Our study suggests that the effects of environmental stressors on social behavior may be unpredictable and dependent on the metric used to measure cohesion, highlighting the need for mechanistic studies to link behavior to the physiology and sensory effects of environmental stressors.

## Graphical abstract

Higher temperature and turbidity acted independently to alter fish social behavior. Fish distribution in the arena became more aggregated in warmer water. With turbidity, wide‐scale interactions were severed resulting in less cohesive groups.

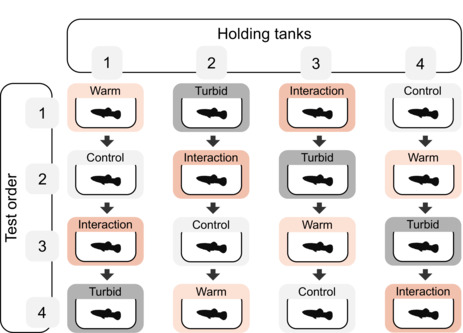

## 1 INTRODUCTION

Shoaling is widespread in fish, having adaptive benefits in predator avoidance, food detection, and reduced energy expenditure through hydrodynamic effects (Conradt & Roper, 2005; Ioannou, Couzin, et al., 2011; Johannesen et al., 2014; Magurran, 1990; Parrish & Edelstein‐Keshet, 1999; Pitcher & Parrish, 1993; Santiago‐Arellano et al., 2021). Collective behavior in shoaling fish involves group formation and maintenance, and relies on the effective transfer of information between individuals. This typically occurs from individual fish detecting and responding to passive social cues in their local environment, such as the location, orientation, and movement of other individuals within their sensory range, which is believed to be dependent on two modalities as the primary sensory inputs (Scott et al., 2023): vision and the lateral line. Vision facilitates attraction and alignment between individuals (Kowalko et al., 2013; Partridge & Pitcher, 1980) and the lateral line modulates the repulsion of near neighbors to avoid collision by detecting changes in water pressure (Faucher et al., 2010).

To gain the benefits of grouping, it is important that collective behavior remains robust by maintaining group cohesion and effective information transfer (Giardina, 2008). However, natural habitats are susceptible to environmental change, and research has indicated that increases in environmental stressors (changes in environmental conditions that cause alterations in a biological response [Orr et al., 2020]) often have negative effects on social behavior (Fisher et al., 2021). Environmental stressors can have anthropogenic causes, which are an increasing threat to global biodiversity, ecosystem functioning, and habitat survivability (Orr et al., 2020). Freshwater habitats are particularly vulnerable due to often being fragmented, yet contribute disproportionately to global biodiversity (Ormerod et al., 2010). Anthropogenic activities such as urbanization and agriculture can cause shifts in water flow, water temperature, eutrophication, turbidity, and levels of pollution within the aquatic environment (Barnett et al., 2008; Landrigan et al., 2018; Vörösmarty et al., 2013). However, while some studies have explored the ecological and physical effects of co‐occurring multiple stressors (Darling & Côté, 2008; Orr et al., 2020) and others have tested how collective behavior changes with single stressors (Bartolini et al., 2015; Herbert‐Read et al., 2017), far fewer studies have explored how social behavior is impacted by multiple stressors (Fisher et al., 2021; Ginnaw et al., 2020; Kuruvilla et al., 2022).

Environmental stressors can interfere with social behavior through masking (van Oosterom et al., 2016) by which the transmission and reception of socially‐relevant information are diminished (McNett et al., 2010). With limited visibility under dark or turbid conditions, shoal cohesion may be reduced due to less available visual information on the position and movement of group members (Chamberlain & Ioannou, 2019; Chaput et al., 2022; Ginnaw et al., 2020; Pitcher & Turner, 1986). Other environmental stressors may not mask visual cues but can affect collective behavior by causing distraction (Herbert‐Read et al., 2017) or direct physiological stress, which can be measured by cortisol secretion (Wysocki et al., 2006). In such situations, less attention may be focused on other individuals in the group as more effort is required for tasks such as predator detection (Sohel & Lindström, 2015). These stressors can include anthropogenic noise (Crovo et al., 2015; Herbert‐Read et al., 2017; Williams et al., 2015; Wysocki et al., 2006), hypoxia (Domenici et al., 2017; Israeli, 1996; Moss & McFarland, 1970; Shoji et al., 2005), increased dissolved carbon dioxide (Bignami et al., 2013, 2014; Duteil et al., 2016; Jutfelt & Hedgärde, 2013), and increased temperature (Johnson et al., 1998; Schulte, 2007; Sylvester, 1972; Weetman et al., 1998, 1999).

With climate change, a current and prominent threat, water temperatures in aquatic habitats are increasing. The IPCC (Stocker et al., 2013) projected a 2–4°C increase in overall global temperatures by the end of the century. Aquatic species are particularly at risk, as water sources store much of the heat associated with climate change. Temperature significantly impacts fish behavior and physiology (Bartolini et al., 2015; Cossins & Bowler, 1987). Fish can be more active and faster swimming in warmer waters due to direct effects linked to the mechanics of muscle kinematics (James, 2013), or by indirect effects, such as increased foraging behavior due to a higher metabolic rate. With heightened activity and energy levels in warmer water (Biro et al., 2010; Holt & Jørgensen, 2015; Weetman et al., 1998), fish may be able to shoal more effectively due to improved swimming abilities. Predatory fish can exhibit higher attack rates with increased water temperature (Krause & Godin, 1995; Persson, 1986; Sylvester, 1972), thus elevated antipredator behaviors such as shoaling in warmer water may be adaptive and a result of physiological changes (Kuruvilla et al., 2022). However, climate change can have variable effects on different species, and the increasing temperature has been linked to a decrease in shoaling in some species (Bartolini et al., 2015; Colchen et al., 2016). For example, as warmer waters hold less dissolved oxygen (Mackay & Fleming, 1969), increasing school volume (i.e., increased spacing between individuals) has been found to be an efficient way to maintain the advantages of shoaling while increasing oxygen flow per individual (Domenici et al., 2017).

Though some aquatic habitats are naturally turbid, many fresh and coastal waters have seen a rapid increase in turbidity due to anthropogenic activity (Mi et al., 2019). Agriculture, deforestation, and urbanization cause eutrophication and sedimentation, resulting in increased turbidity (Davies‐Colley & Smith, 2001). Turbidity reduces visibility via suspended particles scattering light (Utne‐Palm, 2002), and thus impedes reliance on visual cues for aquatic species. This limits both private information (gained directly from the environment) and social information (acquired indirectly from the behavior of other individuals) (Ioannou, Couzin, et al., 2011). It is also suggested that the fragmentation of shoals in turbid waters may not only occur due to a mechanistic visual constraint (Kimbell & Morrell, 2015a) but also due to a lower perceived risk of predation (Sohel & Lindström, 2015). As shoaling is a known antipredator behavior, with reduced risk perception shoals may split as an adaptive behavioral change (Chamberlain & Ioannou, 2019). This is supported by studies that find that fish may be bolder (Engström‐Öst & Mattila, 2008; Robertson et al., 2007), more active (Johannesen et al., 2012; Wishingrad et al., 2015), and less risk‐averse in turbid water (Abrahams & Kattenfeld, 1997). However, others have found increased antipredator behaviors with increased turbidity (Chamberlain & Ioannou, 2019; Leahy et al., 2011; Leris et al., 2022) as reduced visibility may increase the vulnerability of individuals by reducing their ability to detect predators (Sohel & Lindström, 2015). While the antipredator behavior of individual prey in turbid water shows mixed results, a decrease in shoaling in turbid water compared with clear water has been demonstrated repeatedly (Borner et al., 2015; Chamberlain & Ioannou, 2019; Fischer & Frommen, 2013; Kimbell & Morrell, 2015b; Michael et al., 2021).

Most studies have researched the physiological, behavioral, or ecological effects of isolated environmental stressors while other stressors are unmeasured or held constant. However, it is rare for organisms to be exposed to variation in only a single environmental variable (Davis et al., 2018). Individuals will often be exposed simultaneously to several environmental stressors, and these multiple stressors can combine in complex ways (Pistevos et al., 2017). When multiple stressors co‐occur, the response may be dominated by the response to only one of the stressors (known as a comparative effect; Folt et al., 1999), or be the sum of the responses to the stressors in isolation (an additive effect; Ginnaw et al., 2020). However stressors can also interact with one another, where the overall response is greater or less than the sum of the two responses, creating what is known as ecological synergies or antagonism, respectively (also known as nonadditive effects, Côté et al., 2016; Darling & Côté, 2008). It is important to carry out controlled experiments explicitly evaluating the interaction of multiple stressors as such responses are difficult to predict from studies of stressors in isolation (Juvigny‐Khenafou et al., 2021).

Here we aimed to examine how the environmental stressors of temperature and turbidity impact shoal cohesion in Trinidadian guppies (*P. reticulata*) both independently and in combination. Turbidity and temperature were chosen as they have the potential to disrupt the same behaviors via different modalities. Turbidity would impact the visual abilities of the guppies (Borner et al., 2015), and temperature would impact their metabolism and activity (Bartolini et al., 2015). Guppy populations in Trinidad are exposed to varying levels of temperature (23–32°C, Reeve et al., 2014) and turbidity (0–>300 NTU, Magurran & Phillip, 2001). Both environmental parameters can affect social dynamics and shoaling tendencies with conspecifics (Weetman et al., 1998). Though studies have explored the thermal ecology of guppies (Reeve et al., 2014), and other effects of turbidity on their social behavior (Borner et al., 2015), the combined effects of these stressors on the guppy's shoaling behavior are unknown. Shoaling is likely to be particularly important in guppies as their collective behavior varies with the level of predation risk in their environment (Huizinga et al., 2009; Seghers, 1974; Song et al., 2011; Wade et al., 2020). Shoals of guppies were tested in either warmer water (+7°C), turbid water, both warmer and turbid water, or control (clear water at a temperature that matched their housing conditions) treatments in a fully factorial design. From previous studies, we predict guppies to respond to increased turbidity by being less aggregated and cohesive (increased near‐neighbor distance and average inter‐individual distance) (Borner et al., 2015). Other studies (Munoz & Blumstein, 2012; Reeve et al., 2014; Weetman et al., 1999) report that higher temperatures (>26°C) in the absence of predators result in guppies reducing their activity levels, swimming speed and preferring smaller shoals. Consequently, if both temperature and turbidity negatively affect shoal formation and maintenance, we hypothesize that shoaling will be strongest in clear, cooler (ambient temperature) water and reduced in warmer and turbid water. A major aim of the study was to test whether the effects of the two stressors are independent or instead interact synergistically with one another to change shoaling behavior beyond the additive effects of each individual stressor.

## 2 METHODS

### 2.1 Experimental subjects and housing

Trinidadian guppies (*P. reticulata*) were originally caught under license from the Guanapo river in Trinidad by researchers at the University of Oxford, UK, and after a minimum of three generations, transported to the University of Bristol. Guppies were reared in clear water, and only lab‐reared descendants of the original stock were used in the experiment to reduce heritable and developmental effects from the environmental conditions where the ancestral fish were originally caught (Ehlman et al., 2015). The population of mixed‐sex adult guppies (*n*_ind_ = 720) was equally divided and individuals were haphazardly allocated to one of four holding tanks. Each tank (200 L) had independent filtration (provided by an external Eheim 2217 filter), aeration, and heating. The holding tanks were enriched with artificial plants and rolled up the plastic mesh that acted as refuges for fry and smaller guppies. 20%–50% water changes were performed every 4 days in each of the tanks. Water quality parameters (ammonia, nitrate, nitrite, and pH) were monitored once a week. The water in the holding tanks was always clear, and the light regime was kept on a 12:12 h light:dark cycle. Fish were initially held at an average water temperature of 22°C ± 0.57 SD but for the tanks scheduled to be tested in the treatments that included an elevated temperature, this was increased to 29°C ± 0.48 SD for 72 h before the start of these trials. This increase in temperature is representative of the temperature range recorded in the guppy's native rivers (Magurran & Phillip, 2001). The fish were fed after testing each day with TetraMin Flakes (Tetra). The experimental trials were conducted in February and March 2021.

### 2.2 Experimental treatments

**FIGURE 1:**
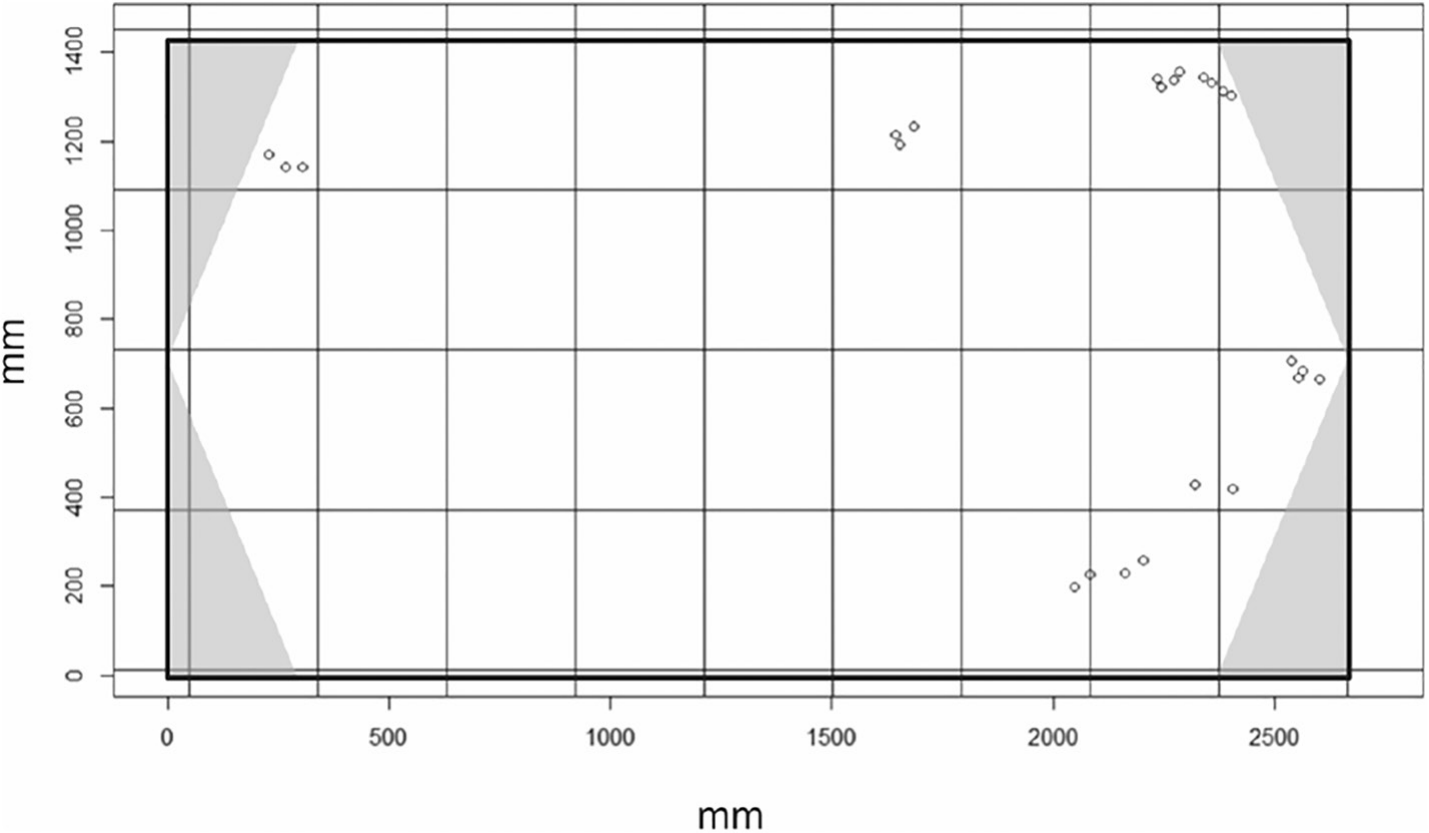
The experimental arena. The gray areas in the corners represent the refuges and open circles are an example of fish positions in a single frame. The thin solid lines depict the reference grid superimposed during the data analysis used to measure the index of dispersion as the variance in the number of fish per grid cell divided by the mean number per grid cell.

Treatments used either clear water (mean ± SD = 0 ± 0.10 NTU) or turbid water (5 ± 0.78 NTU) within the experimental tank (L × W × H = 2550 × 1400 × 100 mm, Figure 1). The level of turbidity selected for this experiment allowed for accurate measurement of fish positions within the arena. Additionally, while previous studies of shoaling have typically used more turbid water (Borner et al., 2015; Michael et al., 2021), they also used smaller experimental arenas where visual contact between individuals is likely to be maintained unless turbidity is high; the experimental arena used in our study was much larger relative to the size of the fish (Figure 1), allowing for the effects of a lower level of turbidity to be tested. The level of turbidity in the turbid treatment is in the medium‐to‐low range of Trinidadian streams (0–60 NTU, Ehlman et al., 2018). On days of the treatments that included turbidity, a clay‐water solution was prepared by mixing 1.5–2 g of white, powdered kaolin clay with 2 L of water from the experimental tank. Once dissolved, the solution was poured into the experimental tank and the water within the tank was mixed thoroughly before each trial. Turbidity was measured using a turbidity meter (Thermo Scientific Orion AQUAfast AQ3010) before and after each turbidity trial. Three 5 mL water samples were collected from three different locations within the experimental tank and their turbidity measurements were averaged for each trial, while for the clear treatment, turbidity was measured at the beginning and end of the testing day. The experimental tank was emptied and cleaned to remove all clay residue and refilled with fresh water before any clear water treatment trials.

The temperature treatments were either the temperature the guppies were initially housed at (mean ± SD = 22 ± 0.57°C) or an increased temperature (29 ± 0.48°C). These temperatures were selected as although research on guppies has found an increase in shoaling behavior in warmer water (26°C compared with 22°C) when a predator is present (Weetman et al., 1999), once a threshold temperature is reached (~30°C), guppies become less active (Munoz & Blumstein, 2012), which might impair their shoaling abilities. Water temperature was manipulated with submersible thermometers with built‐in thermostats (1 × 500 W, 3 × 300 W, 2 × 25 W), which were housed within the refuges provided in the experimental tank's corners by overhanging black PVC triangles (Figure 1). Pilot studies were carried out prior to the experiment to ensure that the water temperatures at the centre of the experimental tank and inside of the refuges were within 1°C. Before and after each trial, the water temperature in both the holding tank and experimental tank was measured using a thermometer to ensure the water was at the appropriate temperature for the allocated trials that day. Trials were run between 10:00 and 18:00 allowing for 2 h of light before the start and after the end of each test day.

### 2.3 Experimental protocol

**FIGURE 2:**
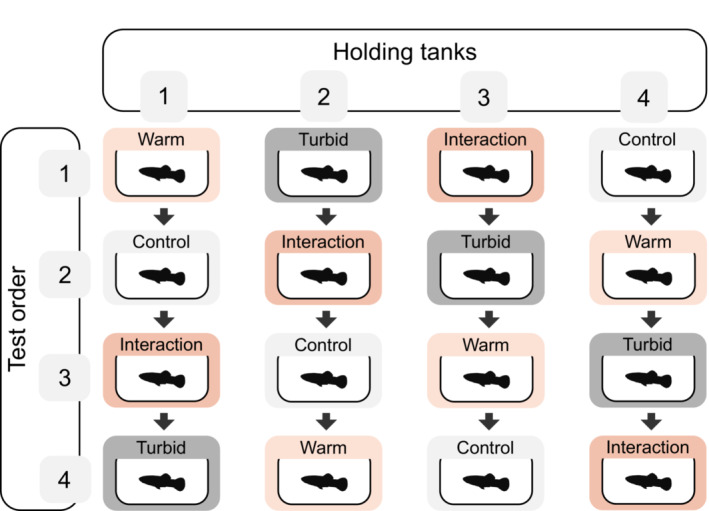
Schematic of experimental protocol detailing the balanced crossover Latin square design for treatment order, where: Control = 0 NTU + 22°C, Turbid = 5 NTU + 22°C, Warm = 0 NTU + 29°C, and Interaction = 5 NTU + 29°C. The Latin Square design ensures that the treatment test order for each holding tank is unique, while each treatment appears once and only once in each test order position (1–4).

Fish from each of the four holding tanks were tested once in each of the four treatments using a repeated measures design over 16 days (Figure 2; *n*_trial_ = 94; two trials (day 4, trial 5 [a control treatment], and day 7, trial 4 [a turbidity treatment]) were excluded as the camera malfunctioned during filming). The treatments were control (0 NTU, 22°C), turbid condition (5 NTU, 22°C), increased temperature (0 NTU, 29°C), and both turbidity and increased temperature (5 NTU, 29°C). This allowed for the effects of turbidity and temperature to be tested both in isolation and in combination. The order of treatments was arranged in a balanced crossover Latin square design (Figure 2) to reduce order effects. For each holding tank, the control and increased temperature treatments were alternated to ensure that the fish were not kept at an elevated temperature for more than 3 days. Each day, six trials were carried out, with each trial testing 29.96 ± 0.76 SD fish caught from the same holding tank (26–32 fish per group; the small variation was due to human error in netting the fish to the arena). Fish already tested that day were held in a temporary holding tank (which matched the conditions of their holding tank of origin) to ensure each guppy was only tested once per day, and hence once per treatment.

For each trial, the ~30 fish were haphazardly caught with hand nets from the holding tank and were gently released in the middle of the experimental arena. Having a large sample of individuals from each holding tank (~30 fish) and testing almost all fish from each holding tank per day allowed for low between‐group variance in sex ratio and other sources of inter‐individual variation. Also, as the individuals forming the test groups from a single holding tank varied trial‐to‐trial and from one test day to another, differences in familiarity between tested fish are likely to be minimal (Cattelan et al., 2019; Heathcote et al., 2017). The time individuals spent in the experimental arena was also short relative to the time spent in the holding tanks, as each trial comprised of 5 min acclimatization time followed by 10 min of recording. The trials were filmed from above with a centrally placed camera (GoPro Hero6) positioned 140 cm above the centre of the tank. Trials were recorded in 2.7 K resolution (2704 × 1520 pixels), in linear mode, and in a frame rate of 30 frames per second.

### 2.4 Data processing and behavioral parameters

From each of the 10‐min videos, one frame every 10 s was extracted with MATLAB R2019a (*n*_frames_ = 6032). The *XY* coordinates of each fish not under a refuge in each frame were manually recorded using ImageJ version 1.53 h (Schneider et al., 2012). Using R Studio version 1.3.1093, behavioral parameters for refuge use and social cohesion were calculated for each frame from this coordinate data. To measure refuge use as a proxy for risk‐taking tendency (Harris et al., 2010), we used the number of visible fish in the experimental arena (i.e., fish not under the refuges), averaged (mean) across all frames in a trial. To measure group cohesion at a local scale, we used the mean nearest neighbor distance (NND), calculated as the minimum distance between each fish and the closest other fish, with the mean NND for all fish in one frame then averaged (again using the mean) for all the frames in a trial. At a broader, global scale, aggregation was calculated as the mean inter‐individual distance (IID) from the mean of the distances between each fish and all the other fish visible in the arena at each frame, again averaged (using the mean) for all fish per frame and then all frames per trial. As an additional global measure of aggregation, the distribution of all visible fish within the experimental arena was calculated by superimposing a grid over the arena (Figure 1) and calculating the number of fish within each grid cell (Perry & Hewitt, 1991). The index of dispersion was then calculated as the ratio of the variance over a mean number of occurrences (i.e., the variance in the number of fish per grid cell divided by the mean number of fish). This ratio per frame was then averaged (mean) across all frames per trial. The start time of each trial was also used to calculate the minutes since midnight, providing a measure of the time of day for each trial so that natural diel variation in fish activity levels could be accounted for.

### 2.5 Statistical analysis

Each behavioral parameter (i.e., the number of visible fish, mean NND, mean IID, and index of dispersion) was analyzed as a response variable in a separate linear mixed model (LMM) using the lme4 package version 1.1‐21 (Bates et al., 2015) in R Studio version 1.3.1093. The turbidity treatment (clear or turbid water), temperature treatment (ambient or increased temperature), repeated testing (day 1–4; repeated testing has been shown to decrease collective behavior in fish shoals [MacGregor & Ioannou, 2021]), number of fish differing from the target of 30 (30 ± 4 fish per trial), and minutes since midnight were included in the models as fixed effects. The holding tank was included as the random effect to account for the repeated measures design. All models initially included an interaction term between the temperature and turbidity treatments, as well as their main effects. Nonsignificant interactions terms between temperature and turbidity were removed from the final models based on likelihood ratio tests (function “drop1” in the “lme4” package in R; Bates et al., 2015), and the effect size of each interaction term was calculated according to Brysbaert and Stevens (2018). The distributions of the residuals for each model were normal, confirmed by using QQ plots. Homoscedasticity (homogeneity of variances) was confirmed by plotting the residuals against the fitted values.

### 2.6 Ethical note

All methods and procedures were performed in accordance with ASAB/ABS guidelines for the treatment of animals in behavioral research. The research was approved by the University of Bristol Animal Services Ethical Committee (UIN/21/003). In this research project, it was essential to use live animals to understand the effects on their group behavior. Care was taken to minimize the handling and stress of the study subjects. Turbidity treatments were limited to concentrations of 5 ± 0.78 NTU, and temperature treatments to a limit of 29 ± 0.48°C. The white kaolin clay used for the turbidity treatments is commonly used for behavioral experiments (Ferrari et al., 2010). Less than 1 g of clay powder per liter of water was used (Johannesen et al., 2014), which is 7 times below the level of turbidity shown to affect gill histopathology in larval fish (Ljubobratović et al., 2019), and is representative of ecologically realistic conditions for guppies (Magurran, 2005; Magurran & Phillip, 2001). Water temperature changes were made gradually and within a recommended range to not induce physiological stress with temperatures near the maximum tolerance for the guppies (Shah et al., 2017). After their use in the experimental treatments, the fish were rehoused in the facilities at the University of Bristol for further experiments.

## 3 RESULTS

There was no evidence for any significant interactions between turbidity and temperature in predicting the behavioral responses (number of visible fish: *p* = .63, *d* = 0.37; mean NND: *p* = .56, *d* = −0.21; mean IID: *p* = .08, *d* = −0.67; index of dispersion: *p* = .73, *d* = 0.12). The minutes since midnight (i.e., the time of day) also showed no significant effects (Table 1). The difference in a number of fish per trial had a weak effect on the index of dispersion, where the presence of more fish in the arena resulted in higher aggregation. The difference in a number of fish included in the arena was nonsignificant for all other response variables. Repeated testing had a significant effect on all three social behavioral parameters, indicating that over the days of testing, fish acclimatized to repeated exposure to the protocol and experimental tank. Mean NND and mean IID increased with repeated testing over days, while the index of dispersion decreased, all indicating that shoaling tendencies decreased over time. The number of visible fish also increased with repeated testing; this reduced refuge use is expected as fish acclimatized to the test protocol and arena.

**TABLE 1:** Model structure and output of the four behaviors measured.

Model structure	Random effects			Fixed effects		
		Variance	SD		LRT	*p*‐Value
Number of visible fish ~ Temperature + Turbidity + Minutes from midnight + Test order + *N* of fish difference + (1|Holding Tank)	Holding tank	1.912	0.409	Turbidity	0.035	.850
				Temperature	21.105	**<.001**
				Minutes from midnight	0.518	.471
				Test order	14.173	**<.001**
				*N* of fish difference	0.099	.752
Mean IID ~ Temperature + Turbidity + Minutes from midnight + Test order + *N* of fish difference + (1|Holding Tank)	Holding tank	80.810	24.550	Turbidity	9.560	**.001**
				Temperature	0.687	.407
				Minutes from midnight	0.065	.798
				Test order	64.707	**<.001**
				*N* of fish difference	3.478	.062
Mean NND ~ Temperature + Turbidity + Minutes from midnight + Test order + *N* of fish difference + (1|Holding Tank)	Holding tank	11.298	3.114	Turbidity	2.933	.086
				Temperature	6.074	**.013**
				Minutes from midnight	1.747	.186
				Test order	43.087	**<.001**
				*N* of fish difference	0.079	.778
Index of dispersion ~ Temperature + Turbidity + Minutes from midnight + Test order + *N* of fish difference + (1|Holding Tank)	Holding tank	0.629	0.308	Turbidity	6.252	**.012**
				Temperature	9.906	**.001**
				Minutes from midnight	0.893	.344
				Test order	45.547	**<.001**
				*N* of fish difference	4.358	**.036**

Abbreviations: LRT, Likelihood Ratio Test; NND, nearest neighbor distance; SD, Standard deviation of random effects. Bold *p*‐Values highlight statistical significance.

**FIGURE 3:**
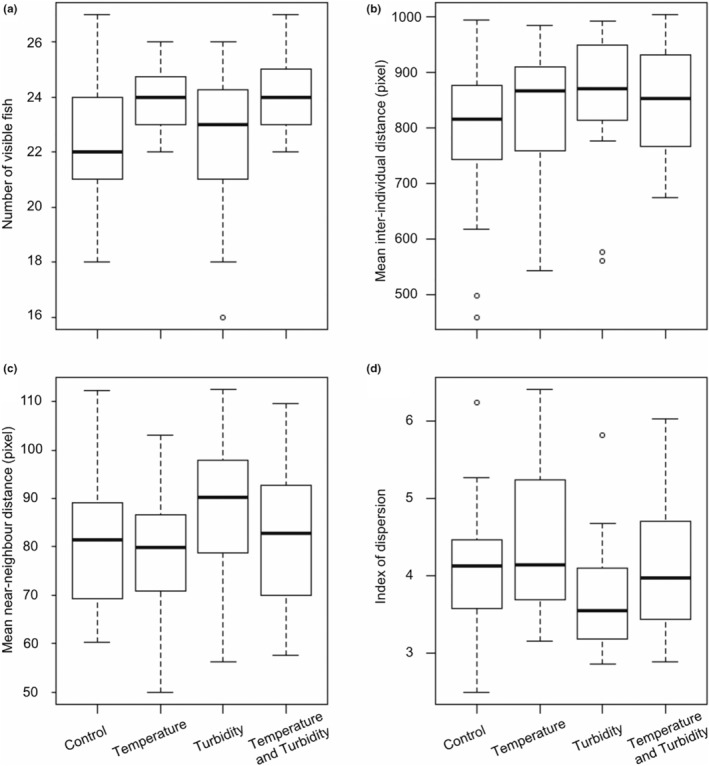
Behavioral variables measured in the four experimental treatments. (a) Number of visible fish in the experimental tank, i.e., fish outside of the refuges as a measure of refuge use. (b) Mean distance between nearest neighbors. (c) Mean inter‐individual distance. (d) Index of dispersion. Values for each frame are averaged (mean) across all frames in a trial (*n*_trial_ = 94). Horizontal black lines within the boxes represent the median value across trials. The edges of the boxes represent the lower (25th percentile) and upper (75th percentile) quartiles. The whiskers extend from the most extreme data point by 1.5 × the interquartile range. The black circles represent outliers.

After controlling for changes over repeated testing by including the day of testing as a main effect in the models, when turbidity and temperature treatments were tested as main effects, there was evidence for both significant and nonsignificant effects on the behavioral response variables. In the warm temperature treatment, fish were more aggregated within the arena (increased index of dispersion, Figure 3d) and more fish were visible (Figure 3a), indicating reduced refuge use by the fish in warmer water. The mean NND decreased in the temperature treatment (Figure 3c), i.e., fish were closer to their nearest neighbor in warmer water; however, temperature did not affect mean inter‐individual distance (IID, global aggregation, Figure 3b). Under turbid conditions, the mean inter‐individual distance (IID) increased, so that the fish were further apart in turbid water. The index of dispersion decreased in the turbidity treatment, also indicating that the fish were less aggregated (i.e., more evenly distributed in the arena) in turbid water. However, fish were not further apart from their nearest neighbor, as the mean NND did not change significantly in the turbidity treatment. The number of fish visible in the arena also did not change significantly in the turbidity treatment. These results suggest that in turbid water, small‐scale distances between near neighbors could be maintained (NND), however, beyond a certain distance, sensory contact between individuals was lost, increasing the average distance among the fish (IID and index of dispersion).

In all combinations of the treatments, the index of dispersion was above one (Figure 3d), indicating that the fish were more aggregated than expected from a random distribution, where a random distribution is expected from no social interactions between the fish.

## 4 DISCUSSION

This study assessed the combined effects of multiple stressors on fish refuge use and group cohesion, using ecologically realistic changes in water temperature and turbidity as focal environmental parameters. As no evidence for any significant interaction terms between the stressors was found in the statistical models, the effects of increased water temperature and turbidity appear to be independent. While increasing the warmer temperature used in this experiment would have physiological effects on the fish that could risk their health and welfare (Shah et al., 2017), higher levels of turbidity than 5 NTU would not have these negative effects and remain ecologically realistic for the guppy (Magurran & Phillip, 2001). Using more turbid water may have led to interactions between turbidity and temperature being found. The effect of increased temperature seemed to dominate the response to increased turbidity for refuge use, while for other behavioral metrics, we see an additive response where the median response in the combined treatment falls within the overlapping responses for increased temperature and turbidity. Similarly, the effects of acoustic noise and darkness were also found to independently impact on the collective motion of three‐spined sticklebacks (Ginnaw et al., 2020). As independent effects, the stressors affected different characteristics of guppy behavior. In warmer waters, guppies reduced refuge use, demonstrating an increase in boldness (Biro et al., 2010; Forsatkar et al., 2016; Gomez‐Maldonado & Camacho‐Cervantes, 2022; Lukas et al., 2021). We observed an increase in shoaling at the higher temperature as measured by the local‐scale NND and one of the global scale measures (the index of dispersion), although no effect on the other global measure of cohesion, the mean inter‐individual distance, was found. Previous work has also found that fish form tighter shoals at higher temperatures (Krause & Godin, 1995; Kuruvilla et al., 2022; Pitcher & Parrish, 1993; Pritchard et al., 2001; Weetman et al., 1998, 1999). This may be a response to increased risk perception, as higher temperatures also increase predators' metabolic requirements and speed (Domenici et al., 2019; Ohlberger et al., 2012), although this is not supported by our experiment as here the measure of risk perception used (refuge use) was reduced at higher temperatures, suggesting that the fish were bolder. As boldness can be positively or negatively associated with social tendencies (Gartland et al., 2022), further work could explore how the effects of warmer temperatures on boldness and aggregation are related; for example, being more social may allow the fish to use refuges less (Harcourt et al., 2009; Santiago‐Arellano et al., 2021).

In the turbid water treatment, visual information between shoal mates was likely to be disrupted, explaining the reduced cohesiveness as measured by both the mean inter‐individual distance and the index of dispersion. The distance between a fish and its nearest neighbor (the NND) was, however, unaffected by both temperature and turbidity, suggesting that close‐range interactions could be maintained under our test conditions. This could be due to neighbors being visible when close even in turbid water, and/or because guppies use their lateral line to maintain NNDs. The lateral line is effective only at close distances (e.g., a few body lengths, Montgomery et al., 2013; Pohlmann et al., 2001) but would be expected to be unaffected by turbidity (Rowe et al., 2003). The study by Faucher et al. (2010) does suggest that the lateral line is used more to avoid collisions between close fish rather than the attraction between them, suggesting that vision may be the sensory system maintaining close NNDs in turbid water in our study. As the turbidity level in our study was moderate at 5 NTU, effects on close‐range interactions may have been observed at higher levels of turbidity, as used in previous studies (Ehlman et al., 2018) and as can be observed in natural systems (Liley & Luyten, 1985); such a trend would support the use of vision rather than the lateral line. At a larger scale of social interaction, however, turbid water was found to increase the average distances between all fish (the IID) and guppies were more evenly distributed across the arena even with the moderate level of turbidity used in our study. Turbid water therefore acts as a visual barrier for fish, which are further apart, ultimately reducing the overall aggregation of the population.

Shoaling is a widespread and effective antipredator behavior (Ioannou, 2021; Magurran, 1990). In conditions where shoaling fish are prevented from maintaining cohesive groups, such as in turbid or dark water, one would expect nonsocial antipredator behaviors, such as refuge use, to increase (Chamberlain & Ioannou, 2019). In this study, however, guppies did not show an increase in antipredator behavior as measured by refuge use in turbid water. The fish in this experiment may instead have perceived the turbidity as providing refuge (Gregory, 1993), counteracting the reduced ability to maintain cohesive shoals. Despite increasing the conspicuousness of the prey group in clear water, aggregation is usually successful in increasing prey survival after initial detection by a predator (Li et al., 2022). In turbid water, however, aggregated prey has been found to have a lower survival rate compared with dispersed prey, even if initial detection is delayed (Ioannou, Bartumeus, et al., 2011; Johannesen et al., 2014). Other studies have, however, shown evidence of fish exhibiting less risk‐averse behaviors in turbid conditions. For example, in turbid conditions, fathead minnows (*Pimephales promelas*) were observed to increase their use of more risky areas (Abrahams & Kattenfeld, 1997) and sticklebacks showed a decline in refuge use and escape responses (Sohel & Lindström, 2015).

There is some evidence that climate change has affected freshwater species and their distribution over the past decades, with species in colder regions being mostly negatively affected, while species in warm regions exhibit range expansion into higher latitudes and altitudes (Buisson et al., 2010; Heino et al., 2009). However, it is difficult to draw broad patterns as the literature is sparse, with freshwater fish and tropical systems being grossly underrepresented (Dodds et al., 2019; Reid et al., 2019). Investigating multiple stressors is an emerging area of research, with their ecological and behavioral effects attracting more attention in recent years (Côté et al., 2016; Matthaei & Lange, 2017; McBryan et al., 2013; Orr et al., 2020). Freshwater habitats are especially vulnerable to co‐occurring stressors because they are often close to, and accessible by, humans. Freshwaters are frequently modified by manmade structures (e.g., weirs and dams), extensively exploited for services (e.g., clean water and recreational use), and they become a receptacle for pollutants from adjoining land and infrastructure (e.g., sewage, pesticides, and livestock). This intense anthropogenic pressure is impacting freshwater habitats, which are fragmented and harbor high levels of biodiversity but with many species those have relatively lower dispersal potential compared with their marine and terrestrial counterparts, causing freshwater species to remain isolated and unable to relocate to avoid fluctuations in environmental conditions (Dudgeon, 2019; Dudgeon et al., 2006). For example, some salmonids have been found to respond well to land use change in cooler months but not when experiencing thermal stress during maximum temperatures in summer, which was probably due to the lack of access to suitable spawning grounds caused by both drought and increased sediment siltation (Murdoch et al., 2020). It has been reported that up to three‐quarters of studies assessing interactions between multiple stressors resulted in nonadditive responses (Darling & Côté, 2008). Antagonistic interactions, often considered beneficial to the impacted ecosystems, are considered more common responses and the dominant interaction type in aquatic habitats (Darling et al., 2010; Jackson et al., 2016). Although considered rare, synergistic interactions still occur and are reported regularly (Russell et al., 2009; Shahid et al., 2019). These can have severe negative effects on populations and whole ecosystems as they act to amplify feedback (Brook et al., 2008). Therefore, it is vital for future studies to investigate combinations of multiple stressors and their effects. Interactions among multiple co‐occurring stressors are likely to depend on how correlated the responses to individual stressors are, and if the different stressors impact the same behavior (Townsend et al., 2008). Our study has explored the impact of increasing water temperature and turbidity on group cohesion in fish, and despite some significant and contrasting responses to the individual stressors, we observed no significant interactions between temperature and turbidity on any of the behavioral variables investigated. This suggests that the Trinidadian guppy may be well adapted to cope under this level of environmental change; further work is needed to test whether these behaviors may persist when predation threat is also included.

## AUTHOR CONTRIBUTIONS

**Imranah Allibhai:** Data curation (equal); formal analysis (equal); investigation (equal); methodology (equal); project administration (equal); writing – original draft (equal). **Martin J. How:** Data curation (equal); methodology (equal); software (equal); supervision (equal); writing – original draft (equal). **Christos C. Ioannou:** Conceptualization (equal); data curation (equal); formal analysis (equal); funding acquisition (equal); investigation (equal); methodology (equal); project administration (equal); resources (equal); supervision (lead); writing – original draft (equal). **Costanza Zanghi:** Conceptualization (equal); data curation (equal); formal analysis (equal); funding acquisition (equal); methodology (equal); project administration (lead); resources (equal); writing – original draft (equal).

## FUNDING INFORMATION

This research was funded by the UK Research and Innovation, through the GW4 FRESH Centre for Doctoral Training in Freshwater Biosciences and Sustainability, grand code NE/R011524/1 awarded to CZ.

## CONFLICT OF INTEREST STATEMENT

The authors declare no conflict of interest.

## Data Availability

The data that support the findings of this study are openly available in Dryad at https://doi.org/10.5061/dryad.j3tx95xjv.
